# Treadmill-walking impairs visual function in early glaucoma and elderly controls

**DOI:** 10.1007/s00417-024-06530-w

**Published:** 2024-06-10

**Authors:** Rosalie Beyer, Khaldoon O. Al-Nosairy, Constantin Freitag, Francie H. Stolle, Martin Behrens, Gokulraj T. Prabhakaran, Hagen Thieme, Lutz Schega, Michael B. Hoffmann

**Affiliations:** 1 Ophthalmic Department, University Hospitals Magdeburg, Leipziger Str. 44, 39120 Magdeburg, Germany; 2 Department of Sport Science, Institute III, Otto Von Guericke University Magdeburg, Zschokkestraße 32, 39104 Magdeburg, Germany; 3 University of Applied sciences for Sport and Management Potsdam, Olympischer Weg 7, 14471 Potsdam, Germany; 4 Center for Behavioral Brain Research, Magdeburg, Germany

**Keywords:** Visual acuity, Contrast sensitivity, Visual field, Glaucoma, Mobility

## Abstract

**Aims:**

Impaired vision is an additional risk factor in elderly for falls. We investigated the hypothesis that treadmill (TM) walking affects visual function in both healthy elderly and those with early-moderate visual dysfunction due to glaucoma.

**Methods:**

Thirty healthy controls (HC) aged 64–83 years and 18 glaucoma patients (GLA) aged 62–82 years participated in this cross-sectional study. The impact of TM-walking on visual function was assessed binocularly for (i) best-corrected visual acuity (BCVA) with and without crowding effect, (ii) contrast sensitivity (CS), and (iii) and visual field (mean deviation, VF-MD). Visual function was tested while participants were standing or during TM-walking for 2 speed conditions: (i) fast walking at their preferred speed and (ii) walking at a fixed speed of 3.5 km/h.

**Results:**

GLA, most with early-moderate VF loss, performed equally well as HC. Independent of GROUP, an impact of SPEED on visual functions was statistically evident with large statistical effect size for (i) both types of BCVA with a mean loss of 0.02–0.05 logMAR (η^2^ = 0.41) and (ii) VF-MD with mean loss of 1 dB (η^2^ = 0.70), but not for CS.

**Conclusions:**

Here, we introduce a paradigm for the assessment of visual function during walking. We provide proof-of-concept that our approach allows for the identification of walking induced visual function loss, i.e., a deterioration of BCVA and VF-sensitivity during TM-walking in both groups. It is therefore of promise for the investigation of the relation of vision impairment and mobility, ultimately the increased frequency of falls in advanced glaucoma.

**Supplementary Information:**

The online version contains supplementary material available at 10.1007/s00417-024-06530-w.


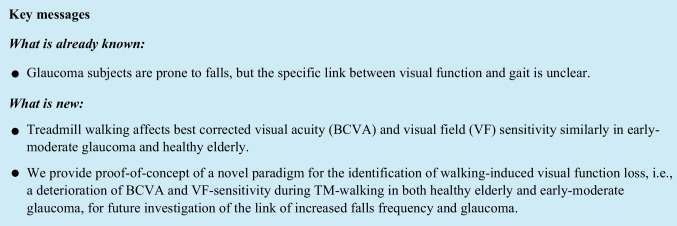


## Introduction

Glaucoma is a leading cause of irreversible blindness worldwide [1, 2]. The term glaucoma summarizes etiologically different disease mechanisms pointing to a common finding, i.e. chronic progressive optic neuropathy with loss of the retinal ganglion cells and corresponding visual field defects [3]. In fact, glaucomatous damage not only affects the eye, but also brain structure and function [4, 5] and leads to functional deficits such as impaired visuo-motor coordination [3]. Trivedi et al. also demonstrated impaired sensorimotor brain connectivity in early glaucoma that was correlated with a reduced balance performance [4]. It is plausible that these glaucoma related brain changes lead to impaired postural control and orientation [4, 6, 7], resulting in an increased incidence of falls [8, 9], which is a leading cause of injury-related death in patients [10].

Gait dysfunction and the risk of falling increase with age. For instance, one third of individuals > 65 years and almost half of people > 80 years fall at least once a year [11, 12]. Being a disease of late onset and age-related progression, glaucoma enhances the likelihood of falling, specifically due to risk factors such as impaired peripheral vision, orientation difficulties and the advanced age of affected individuals [10, 13]. Interestingly, visual function has been reported to be reduced during walking even for participants with healthy vision [14, 15], e.g., visual acuities are reduced during treadmill walking at 1.5 m/s in healthy controls [16].

Vision impairment does not only restrict the mobility of the affected individuals and increases the risk of falling, but also reduces quality of life in glaucoma patients [10, 17]. This motivates investigations to unravel the relation of glaucoma and visual function during gait. An important step for this purpose is the investigation of the effect of gait on visual function in both elderly healthy controls and patients with glaucoma.

In this study, we established a TM-walking paradigm to test the hypothesis that readouts of visual function were influenced by TM-walking in both elderly controls and in participants with glaucoma. We examined the effect of treadmill walking (TM-walking) on visual function by comparing best corrected visual acuity (VA_S_), crowding BCVA (VA_C_) [18], contrast sensitivity (CS) [19], and visual field sensitivity (VF-MD) between elderly participants (≥ 60 years) with healthy vision and with glaucoma. This might provide visual function parameters that are mostly affected during TM-walking and could be employed as sensitive biomarkers of the interaction of locomotion and visual perception.

## Methods

Below included the essential descriptions of methods and for further details, please see supplementary. This study was approved by the Ethics Committee of the Otto-von-Guericke University of Magdeburg, Germany (registration number: 32/18).

### Participants

Forty-eight participants [18 glaucoma patients (GLA) and 30 age- and sex-matched healthy controls (HC)] were recruited in this cross-sectional study.

No significant age difference was observed between GLA (mean age ± SEM: 71 ± 1 year) and HC (71 ± 1 year, *p* = 0.75).

For patient characterization, standard automated perimetry (SAP) was used to assess visual field sensitivities (mean deviation, VF-MD) and optical coherence tomography (OCT) scans were acquired to calculate the average peripapillary retinal nerve fiber layer thickness (pRNFL) and the averaged ganglion cell layer (GCL) and inner plexiform layer (IPL) volume.

## Testing the impact of TM-walking on visual function

The primary aim of the study was to investigate visual function during TM-walking. For this purpose, the experimental setup was limited by two main challenges: (i) the use of testing-schemes that are compatible with a TM-walking setting and (ii) the problem of maintaining the viewing distance for vision testing during TM-walking. To overcome these issues, we (i) selected the appropriate soft- and hardware solutions for vision testing, i.e., the FrACT and Ocusweep®, and (ii) used a distance sensor ("Vivior® sensor").

The subsections below provide detailed descriptions of (a) the viewing distance estimation, (b) the general procedures for testing, and specific approaches for vision testing during TM-walking, i.e. for (c) visual acuity and contrast sensitivity testing, and (d) visual field testing.

**Fig. 1:**
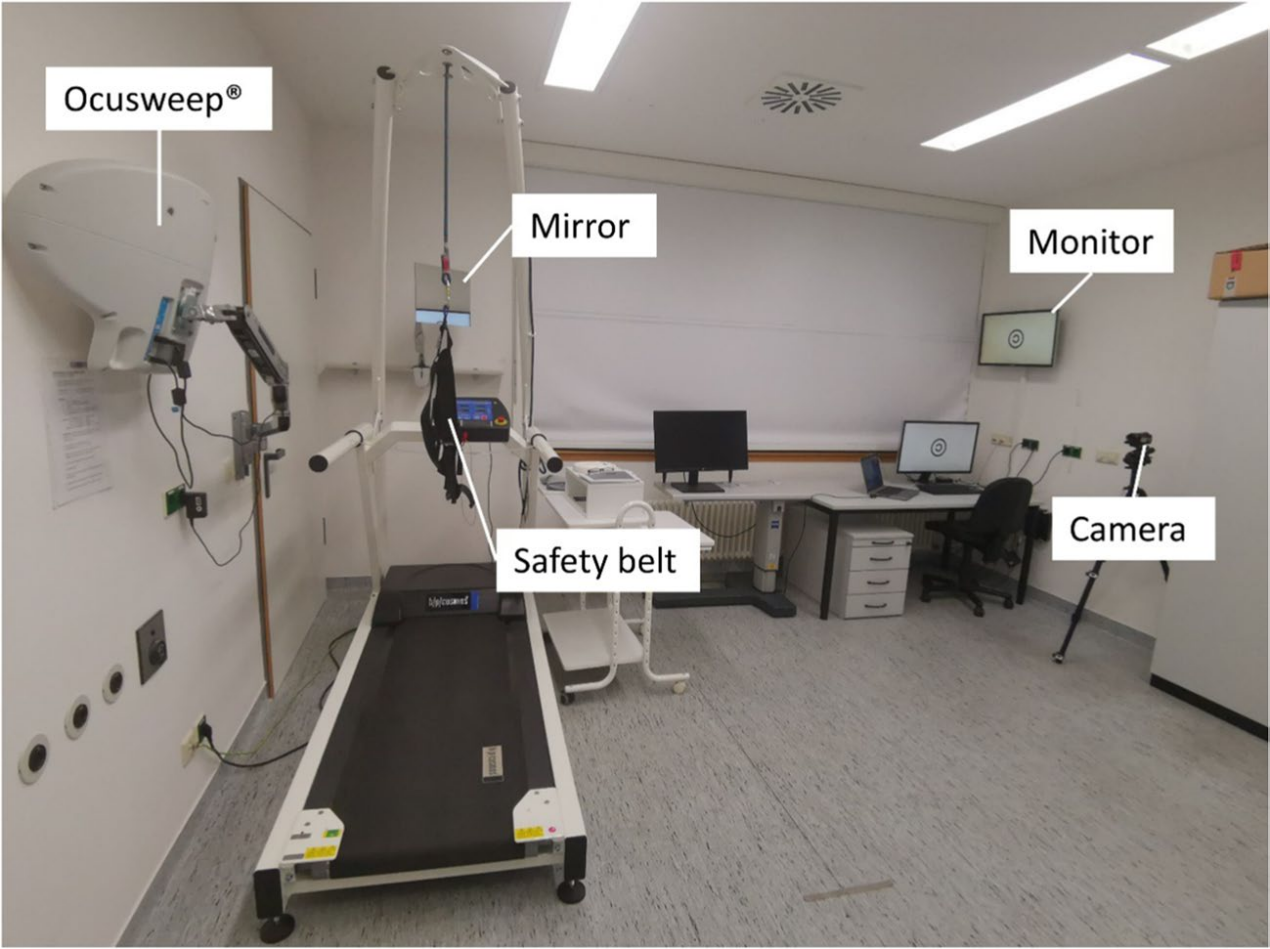
Measurement setup. You can see the treadmill with the safety belt, as well as the mirror through which the test person sees the monitor (on the right wall, here displaying the optotype for crowded VA). The Ocusweep® is attached to the left wall by means of a swivel arm (here 'parked' at the wall, with the back of the device visible). A camera (right) records the measurement

- *Viewing distance estimation.* Only the distance corrected values VA_S/C_ were analyzed, see supplementary for further details. For the CS and VF measurements, no distance correction was applied due to the unavailability of Vivior readouts.
- *General procedure for testing (*Fig. 1*).* In order to determine visual function during TM-walking, visual stimuli were presented to participants [1] during standing (S_0_) or [2] during TM-walking at two speeds: (i) fast walking at a individuals preferred speed (S_self_), and (ii) at 3.5 km/h (S_3.5_). During each of these three conditions, a set of visual function tests were performed after an initial determination of S_self_ on the treadmill. Subsequent vision testing included two repetitions (ABC-ABC) of BCVA testing with and without crowding and CS testing, followed by one repetition of binocularly performed 24–2 VF testing. Prior to the series of measurements, one standard Ocusweep®-VF testing with a proximity sensor control and acoustic prompt for correction was conducted on the treadmill at rest to obtain a baseline value for binocular VF sensitivity (see supplementary Table 1). Total duration of BCVA, CS and VF testing was about 1:15 h.
- *Visual acuity and contrast sensitivity.* BCVA and CS were determined binocularly for the three different speed conditions at a viewing distance of 5 m using FrACT [20], i.e. 8-alternative forced-choice (AFC), in a dimly-lit room. They were reported as logarithmized minimum angle of resolution (logMAR) and Weber contrast (logCS), respectively. BCVA was tested for two conditions, (i) without (VA_S_, a single optotype) and (ii) with crowding (VA_C_, an optotype surrounded by a circle ["©"]). The stimuli were presented on a 28" LED monitor (Samsung U28E590DSL, Dublin, Ireland) and calibrated with Data SpyderX Pro (Datacolor, New Jersey, USA).
- *Visual field sensitivity.* A single repetition of 24–2 binocular VF testing was conducted for each of the three speed conditions with the Ocusweep™ Perimeter (Ocusweep SAP, Ocuspecto Ltd, Turku, Finland). This perimeter enabled testing without head or chin rest under ambient room light at 42.5 cm testing distance controlled via light and proximity sensors, respectively. For the test paradigm, the distance control had to be deactivated to allow for testing during TM-walking. The test stimuli, briefly appearing (100 ms) at different locations across the perimeter, comprised 9 LEDs arranged in a square (5.2 mm = 0.1 log unit smaller than Goldmann IV stimulus) enabling VF testing without refractive correction for near VA > 0.1 logMAR [21, 22]. The participants responded to the stimulus presentations via a remote control. The mean deviation (MD [dB]) was determined for visual field sensitivity assessment. In contrast to the Humphrey Field Analyzer, Ocusweep® uses positive instead of standard negative MD-values to indicate greater visual field loss. For one participant the visual field examination could not be performed for the S_3.5_ condition.

The effect of treadmill walking on systemic vascular parameters, namely the heart rate (pulse), was monitored with a wireless transmitter belt (POLAR Heart RateSensor H9) wrapped around the lower part of the chest coupled with the h/p/cosmos® treadmill setup (h/p/cosmos sports & medical gmbh, Germany). During TM-walking, both groups showed an increase of heart rate during S_self_ & S_3.5_ by 11 beats per minute (bpm) in comparison to baseline static (S_0_) measurements (a mean of 86 bpm), F(1,6, 75,4) =  < 0.001.

## Analysis and statistics

All statistical analysis and assumptions testing, e.g., normality tests, were performed with SPSS 28 (Statistical Package for the Social Sciences; IBM, Armonk, NY, USA) and included repeated measures analysis of variance (RM-ANOVA) for the within-subject factor SPEED and REPETITION and the between-subject factor GROUP as well as post-hoc analyses. Effect size was determined using eta-squared (*η*^2^) value and evaluated after Cohen, i.e., small (0.01), medium (0.06) and large effect (0.14) [23]. The significant p-values reported were corrected for multiple testing using Sidak correction for multiple testing. For further evaluation, the visual acuity loss was calculated by subtracting the results of the S_3.5_ condition from the S_0_ condition, e.g., VA_S_ loss [logMAR] = VA_S_ S_0_ [logMAR]—VA_S_ S_3.5_ [logMAR].

## Results

The sections below describe (a) the effect of TM-walking on viewing distance, a potentially confounding factor for BCVA testing and (b) representative examples for a qualitative overview. This is followed by group analyses of the effect of different TM-walking speeds on (c) the specific vision tests, i.e., VA_S/C_ (corrected for distance variability), CS and VF and (d) visual function loss during TM-walking.

**Fig. 2:**
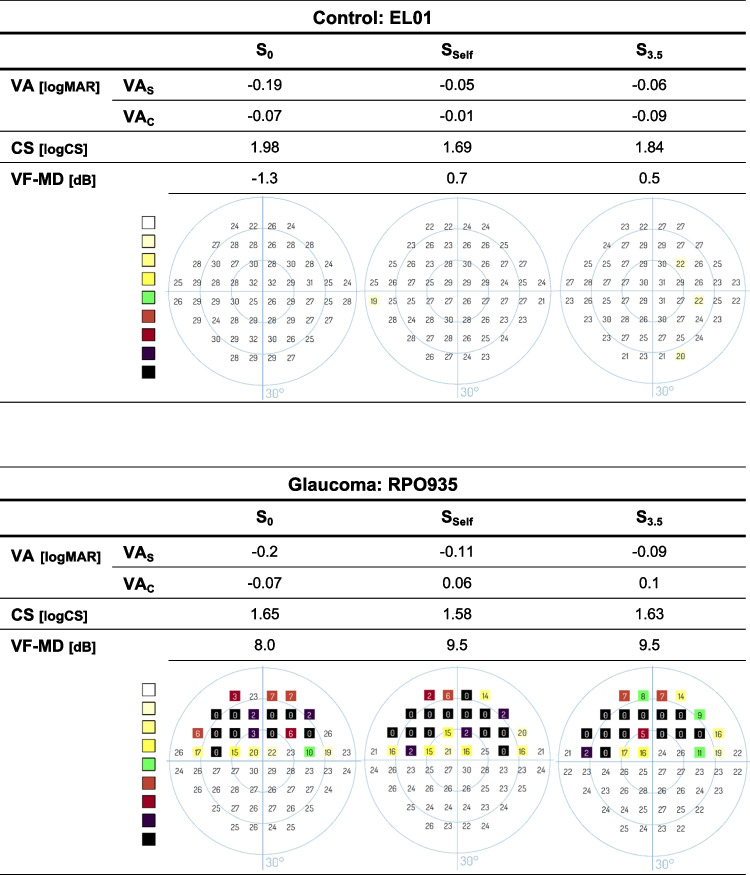
Visual function tests for a HC and GLA for different speed conditions. During TM-walking (S_self_ & S_3.5_), BCVA, with (VA_C_) and without crowding (VA_S_), and VF-MD deteriorated in comparison to S_0_. VA_S_ = visual acuity with a single optotype; VA_C_ = visual acuity with crowding; CS = contrast sensitivity; VF-MD = visual field, mean deviation, S_0_ = static condition, S_Self_ = individual's tolerated fast TM-walking speed, S_3.5_ = 3.5 km/h

- *Impact of TM-walking speed on viewing distance.* As described in methods, the Vivior® sensor allowed the measurement of the viewing distance during visual function measurements for the different speed conditions. In brief, the observed effects of different TM-walking speeds on viewing distance were small compared to the actual viewing distance, i.e., < 5% (16 cm/500 cm * 100%), see supplementary for further details.
- *Representative examples.* A representative data set of a HC and a GLA participant is given in Fig. 2 as an overview of the results obtained for the different visual function tests at the three speed conditions [S_0_, S_Self_, S_3.5_]: (i) VA_S_ and (ii) VA_C_, (iii) CS and (iv) VF. A deterioration of visual function during TM-walking was evident for both groups for VA_S_, VA_C_, and VF, but not for CS. This reflects the overall trend of changes induced by different TM-walking speeds on these visual function tests as detailed below.
- *Impact of TM-walking on visual function tests.* TM-walking induced a comparable effect on the visual function in both groups, as shown in the following sections. A detailed listing of the statistical outcomes of the RM-ANOVAs are summarized in Table 1, i.e., specific factors, F-values, *η*^2^ and post-hoc tests.

**Table 1:** Results of ANOVA and post-hoc analyses

	RM-ANOVA				Post-hoc		
	Factors	F	p	*η*^2^	t-tests	Mean diff	p
*Visual acuity* *[logMAR]*	VA type	F(1,46) = 30.2	** < 0.001**	0.40	-	-	-
	Group	F(1,46) = 1.2	0.275	0.03	-	-	-
	Speed	F(2,92) = 31.7	** < 0.001**	0.41	S_0_ vs S_Self_ S_0_ vs S_3.5_ S_Self_ vs S_3.5_	-0.05 -0.04 0.01	** < 0.001** ** < 0.001** 0.335
	Repetition	F(1,46) = 2.0	0.16	0.04	-	-	-
	VA * Speed	F(2,92) = 3.4	**0.037**	0.07	VA_S_ vs VA_C_ S_0_ S_Self_ S_3.5_	-0.06 -0.04 -0.03	** < 0.001** ** < 0.001** **0.005**
*Contrast sensitivity* *[logCS]*	Group	F(1,46) = 1.4	0.241	0.03	-	-	-
	Speed	F(2,92) = 0.0	0.994	0.0003	-	-	-
	Repetition	F(1,46) = 4.7	**0.035**	0.09	-	-	-
*Visual field-MD* *[dB]*	Group	F(1,45) = 7.0	**0.011**	0.13	-	-	-
	Speed	F(2,90) = 106.3	** < 0.001**	0.70	S_0_ vs S_Self_ S_0_ vs S_3.5_ S_Self_ vs S_3.5_	-0.8^#^ -0.9^#^ -0.1^#^	** < 0.001**^**#**^ ** < 0.001**^**#**^ 0.526^#^

Significant results are highlighted in bold.

^#^Wilcoxon test, with difference of medians

Mean diff. = mean difference

### Visual acuity

**Fig. 3:**
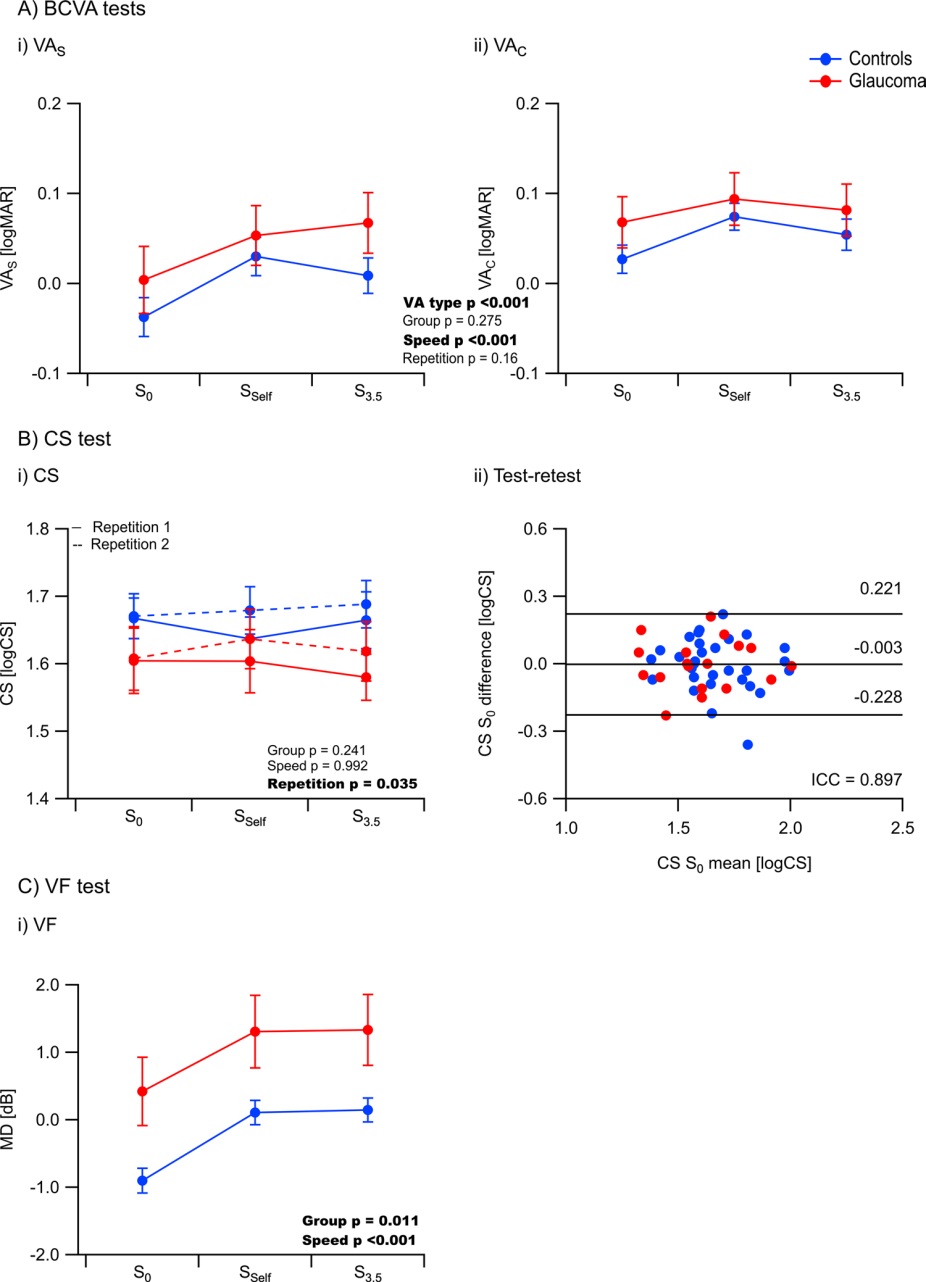
Impact of different TM-walking speeds on visual function tests. **A** BCVA. (i) & (ii) Effect of TM-walking speeds on BCVA (VA_S_ – without crowding, VA_C_ – with crowding); BCVA was worsened during TM-walking compared to standing. **B** Contrast sensitivity (CS). (i) Effect of TM-walking speed on CS; the CS did not change during TM-walking and the repetition effect reached only weak significance. (ii) Bland–Altman diagram of test–retest repeatability of the two repetitions for S_0_ demonstrates good reproducibility over both measurements. **C** Visual field (VF). (i) Effect of TM-walking on VF; reduced VF-sensitivities were evident during walking. BCVA = best corrected visual acuity, ICC = intraclass coefficient, further abbreviations: see Fig. 2

Corrected BCVA of both groups deteriorated significantly during S_self_ (*p* < 0.001) and S_3.5_ (*p* < 0.001) compared to S_0_ by 0.048 ± 0.01 logMAR and 0.038 ± 0.01 logMAR, respectively. As expected, VA_s_ was better than VA_c_ across all speed conditions (*p* < 0.001) (Fig. 3A i, ii). There were no differences between groups for these measures that might be attributed to the largely early nature of damage in GLA.

### Contrast sensitivity

Unlike BCVA measures, CS was unaffected by TM-walking. There was a minor repetition effect (*p* = 0.035, see Fig. 3B i), which was not evident in the post-hoc analysis (*p* > 0.05). In addition, the relative intra-session reliability was high for both CS measurements repetitions indicated by the respective intraclass correlation coefficients (ICC) (S_0_: ICC = 0.90, S_Self_: ICC = 0.82, S_3.5_: ICC = 0.87) (for S_0_: Fig. 3B ii).

### Visual field

Similar to BCVA effects, TM-walking had a significant reductive effect on VF sensitivities, which did not differ between HC and GLA; VF-MD was reduced by 0.95 ± 0.08 dB for S_Self_ and 0.97 ± 0.08 dB for S_3.5_. As expected, VF-sensitivities were worse for the diseased group, GLA, compared to HC (significant group difference, *p* = 0.01), which was evident for all speed-conditions, i.e. S_0_, S_Self_, S_3.5_ (Fig. 3C i).

**Fig. 4:**
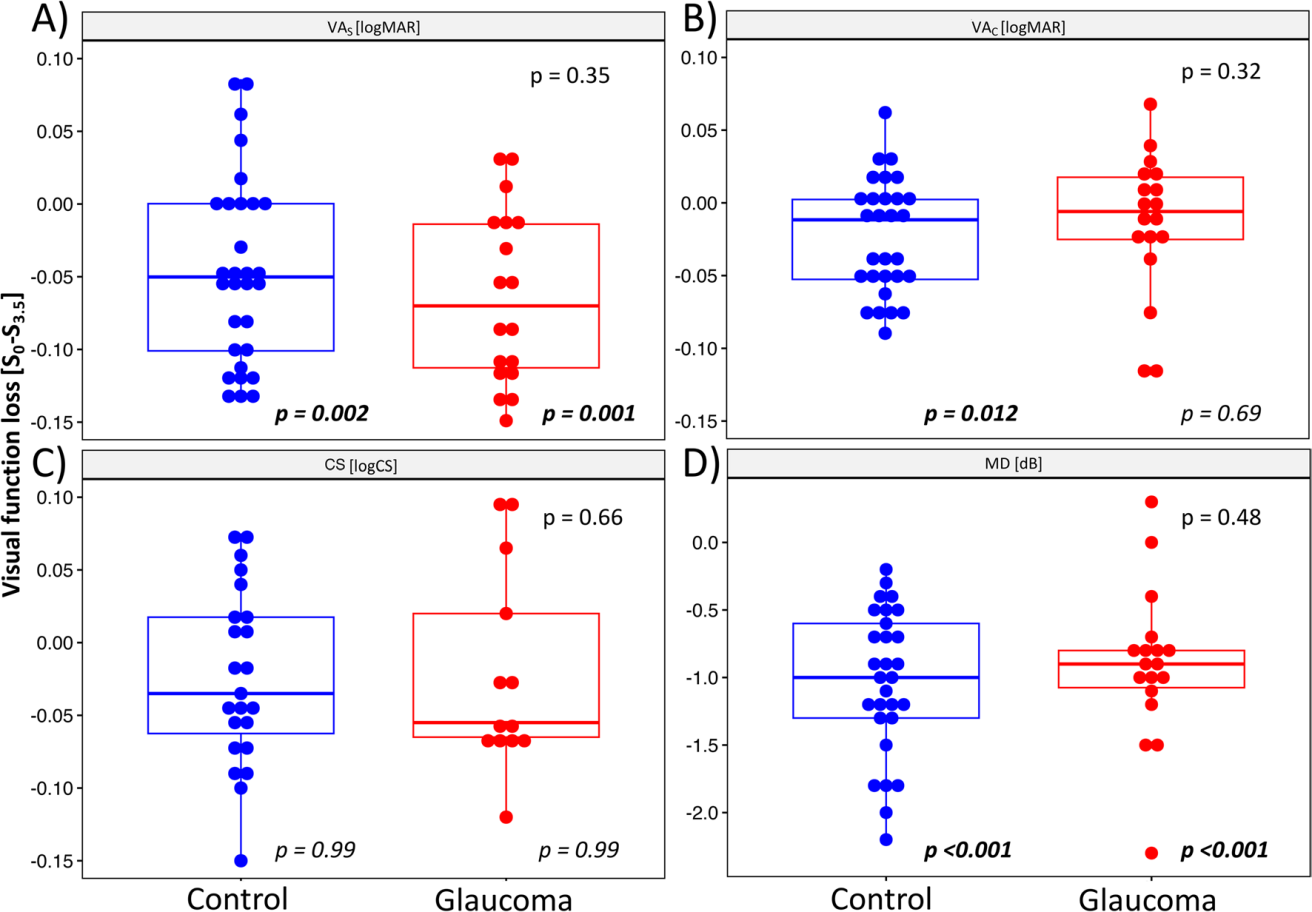
Visual loss associated with TM-walking.p-values not italicized: group difference. p-values in italics: difference from no loss, i.e., 0, for each group separately. Significant results are highlighted in bold. **A** VA_S_ loss. **B** VA_C_ loss. **C** CS loss. **D** VF-MD loss. Significant visual function loss was only evident for VA_S,_ VA_C_ in HC and VF and affected both groups similarly

(d) *Visual loss.* For a comprehensive assessment of the effect of different TM-walking speeds on vision, one-sample t-tests were performed to assess visual function loss during TM-walking for each test [VA_S_, VA_C_, CS, VF] (Fig. 4). Visual loss was calculated by subtracting the test values for S_3.5_ from those for S_0_ and tested for the null hypothesis, i.e., no difference. During TM-walking, visual loss was highest for VA_s_ (HC: *p* = 0.002, GLA: *p* = 0.001) and VF-MD (HC, GLA: *p* < 0.001) for both groups; VA_S_ and VF-MD might, therefore, serve as highly sensitive biomarkers to investigate visual performance during TM-walking and consequently as surrogate biomarkers for falls’ tendency. VA_c_ loss was significantly different from 0 only in HC (*p* = 0.012). There were no significant effects for CS (*p* > 0.05). Group differences for the visual loss were not evident for none of the tests (VA_S_, VA_C_, CS, VF; *p* > 0.05).

## Discussion

### Summary of findings

This study, for the first time, addressed the effect of different TM-walking speeds on visual function of GLA in comparison to age-matched HC. We observed a deterioration of VA_S/C_ and VF-MD during TM-walking compared to standing, but not for CS. Based on our findings, VA_S_ and VF-MD might be potential surrogate biomarkers to identify or assess the risk for falls in GLA and elderly HC. Both HC and GLA demonstrated similar reduction in visual function during TM-walking without group differences, which might be attributed to the early-stage nature of damage in our GLA cohort.

### Vision and gait: GLA vs HC

Vision loss, irrespective of the etiology, is a significant risk factor for falls [24, 25]. This risk is further increased due to diseases like glaucoma [26, 27]. However, the magnitude and the exact influence of glaucoma on the tendency to fall are not sufficiently elucidated. In this regard, the present study aimed to disentangle the effects of different TM-walking speeds on visual function in GLA compared to HC. To our knowledge, no previous studies have reported the impact of different TM-walking speeds on visual function in glaucoma, but the effect of TM-walking on BCVA has previously been investigated in healthy controls with the result of deteriorated BCVA with increasing speed [16, 28]. In fact, our finding of a significant decline of -0.05 ± 0.01 logMAR for VA_S_ and -0.02 ± 0.01 logMAR for VA_C_ at 3.5 km/h corresponds well to the results of Verbecque et al. (2018), who reported a visual loss of BCVA in elderly of the 7th (8th) decade during 3 km/h TM-walking of -0.02 (-0.05) logMAR and during 4 km/h of -0.02 (-0.08) logMAR. In addition, we demonstrated a significant decrease in VF-MD during TM-walking at 3.5 km/h by 1.0 ± 0.1 dB. Given that both groups performed equally during our measurements, it seems that elderly HC and those with GLA exhibit comparable visual function loss during TM-walking compared to standing.

Impairment of vision during TM-walking might imply an increased predisposition to falls in both diseased and healthy elderly groups, due to the relevance of vision for balance and navigation. In glaucoma, previous studies indicated the relevance of visual field loss for gait instability and predisposition to falls, e.g., faster rates of visual field loss were significantly associated with an increased incidence of falls in glaucoma [29]. Further, worse VF defects at baseline were associated with a stronger reduction of walking speeds in elderly glaucomatous participants and hence with gait dysfunction at the end of the follow-up period of 3 years [30]. There was a rapid decline of stride velocity per 5 dB decrease in the integrated binocular visual field sensitivity. Elderly individuals might also be prone to falls as the risk is increasing with aging [10, 12]. Accordingly, visual impairments are strongly associated with two or more falls in older adults. This association was previously reported to be related to reduced VA, VF, and impaired CS and presence of cataract in these individuals [27]. Further, in healthy elderly controls, poor visual performance during TM-walking might be attributed to variable gait patterns in comparisons to younger individuals. For instance, elderly individuals might have abnormal gait patterns, e.g., shorter stride length, affecting the head frequency (head movement per time), which might influence of visual function performance while walking [28, 31]. Further, a longitudinal follow-up of healthy elderly participants reported visual, auditory, vestibular, and brain changes that were correlated with gait and balance measures [32].

### Outlook, clinical relevance and applications

The overall aim of this research is the evaluation of an multimodal exercise-based intervention program especially for glaucoma patients, similar to training programs that have already been shown to reduce the risk of falls in elderly persons [33, 34]. For this purpose, surrogate biomarkers of the intervention success, including those for visual function recovery, need to be identified. In the present study, we demonstrated that the approach presented is viable and that VA_S_ and VF-MD have the potential to serve as biomarkers. Importantly, we demonstrated that in the range of glaucoma induced visual deficiencies of the present study (binocular MD range: 0 to 10), all patients were able to walk on a treadmill equally well as HC. This proof-of-concept encourages the use of the paradigm in a follow-up study in a cohort with more severe visual function deficits.

### Limitations

Glaucoma is widely considered to be an undisputed risk factor for falls, although no greater effect of different TM-walking velocities on GLA compared to elderly HC was observed in the present study. There are several limitations that might have influenced our results. In our study, we mainly tested central vision and included mainly early-moderate stages of glaucoma. In follow-up studies, patients with the loss of the lower VF should be included, as that is considered a specific risk factor for falls [13, 35]. Lack of distance correction of CS and VF measurements is another limitation in this study. While the presented tread-mill-based approach has the benefit to of controlled walking parameters for better comparison amongst participants, it must be noted that the findings of this present study might not reflect physiological changes of visual function during walking indoors and outdoors. Further, we acknowledge that the reported decline of visual function during TM-walking might not be clinically significant, i.e., VF loss of 1 dB or VA loss of 0.02–0.05 logMAR, still it should be noted that the statistical effect size of the reported decline is large (see Methods), η^2^ = 0.4 and 0.7, respectively. Finally, our GLA cohort included only 18 patients due to recruitment difficulties during the corona pandemic and strict inclusion criteria. In conclusion, the extension of the study to a larger sample including more advanced disease stages would be of promise to uncover the effects of different TM-walking speeds on visual function in glaucoma covering a range of disease states.

## Conclusion

In this study, we have found TM-walking to impair visual function for both GLA and HC. In both groups, the effect of TM-walking speed was most pronounced for VA_S_ and VF-MD. Consequently, visual acuity and visual field sensitivity appear to be the most sensitive biomarkers to assess visual performance during locomotion, potentially gait control, and ultimately the risk of falls. At the individual level, these biomarkers might serve to identify persons with enhanced visual function loss during locomotion and thus increased risk of falls. In combination with interventions that address visual impairment related falls, these biomarkers promise readouts of intervention success. Intervention studies might benefit from the application of the TM-walking paradigm introduced in the present study in order to assess the impact of interventions, such as mobility training, on visual perception during locomotion and provide a quantitative readout of the success of therapy.

## Supplementary Information

Below is the link to the electronic supplementary material.

Supplementary file1 (DOCX 297 KB)
