# Supplementary material for: Treadmill-walking impairs visual function in early glaucoma and elderly controls
Source: Graefes Arch Clin Exp Ophthalmol. 2024 Jun 10;262(11):3671–80. doi: 10.1007/s00417-024-06530-w (PMC11584441; doi:10.1007/s00417-024-06530-w)
Supplement: Supplementary file 1 — Supplementary file1 (DOCX 297 KB) [file 417_2024_6530_MOESM1_ESM.docx]

**Supplementary**

**Methods:**

This prospective study was conducted at the ophthalmology department of the Otto-von-Guericke University Hospital from Sep.2020 - Dec.2022, Magdeburg and is registered at DRKS (German Clinical Trial Register, registration number: DRKS00022519). All procedures followed the tenets of the declaration of Helsinki and was approved by the Ethics committee of the Otto-von-Guericke University of Magdeburg, Germany (registration number: 32/18).

**Participants**

All participants underwent complete ophthalmic examinations including best corrected visual acuity (BCVA) testing using the early treatment diabetic retinopathy study (ETDRS) charts, stereovision testing using Lang test, visual field (VF) testing, optical coherence tomography (OCT) imaging and fundus examination (see supplementary Table 1 for details). All participant underwent refraction to correct for testing at 5 meters. Patient with cataract surgery had monofocal intraocular lenses.

Exclusion criteria were: (i) any eye disease affecting the indices of visual functions [e.g., cataract (except incipient stage), ocular trauma history and ocular surgeries (except glaucoma or cataract surgery)], (ii) neurological diseases e.g., stroke, and (iii) diseases that limit the physical performance of the participants including orthopedic diseases, e.g., arthrosis grade ≥ II, musculoskeletal impairments, tendinitis, myositis, joint replacements, rheumatism.

**Healthy controls (HC)**

All the HC included in the study were visually healthy as indicated by a BCVA ≥ 0.8.

**Glaucoma group (GLA)**

Glaucomatous eyes were defined via the appearance of optic disc and a visual field defect, specifically (i) a general increase of cupping defined as vertical cup-to-disc ratio ≥ 0.7, (ii) retinal fiber layer defect and/or a local notching of the optic disc rim, and/or (iii) glaucomatous visual field (VF) defects < -2.0 dB mean deviation. All patients were on IOP-lowering treatment.

**Visual field testing**

For patient characterization, standard automated perimetry (SAP) was used to assess visual field sensitivities (mean deviation, VF-MD) employing the Swedish Interactive Threshold Algorithm 24-2 protocol (SITA-Fast) of the Humphrey Field Analyzer 3 (Carl Zeiss Meditec AG, Jena, Germany).

**Optical coherence tomography**

Optical coherence tomography (OCT) scans were acquired using a spectral domain OCT device employing Glaucoma Module Premium edition (Heidelberg Spectralis®, Heidelberg Engineering, Heidelberg, Germany). Peripapillary retinal nerve fiber layer thickness (pRNFL) was determined from the 3.5 mm scan. Imaging of the macula was performed with a customized scan with a scanning angle of 20° × 20° (6 x 6 mm) centered on the fovea to determine the averaged ganglion cell layer (GCL) and inner plexiform layer (IPL) volume within the early treatment diabetic retinopathy study (ETDRS) thickness circle. OCT measurements were not possible for 2 GLA eyes (one eye missing macular and the other missing pRNFL scan. The missing subjects were not included in the correlation analysis of visual loss vs functional/structural measures of retina.

***Viewing distance estimation.***

For VA_S/C_, CS or VF testing the viewing distance is critical. Via a wall-mounted mirror, participants viewed a monitor presenting the optotypes for VA_S/C_ and CS testing at a distance of 5 m (distance_participant_mirror_ = 1.5 m; distance_mirror_monitor_ = 3.5 m). In order to correct for deviations from the intended viewing distance during TM-walking, induced by head and body movements, we determined the change of the viewing distance during TM-walking. For this purpose, we used infrared distance sensors (10 Hz), i.e. "Vivior^®^ sensor" (VIVIOR AG, Zürich, Switzerland) (1), which were mounted laterally on the participants’ eyeglass frames, as can be seen on the website of the manufacturer (2). The Vivior^®^ sensor used for the distance measurement was intended to point straight ahead, but tended to be misaligned from the horizontal axis, when mounted on the trial eyeglass frames. Therefore, the resulting sensor-based overestimation of the participants distance from the mirror, was compensated by scaling the distance for the tilt angle (i.e., cos(angle)), as determined from video recordings (lateral view of the participants) taken during the measurements: (i) the median distance of the sensor and wall was determined from Vivior^®^ sensor readouts, (ii) the estimated viewing distance to the mirror was corrected based on the tilt angle [cos(angle)], and (iii) 3.5 m (distance from the mirror to the monitor) were added to obtain the complete real viewing distance. Subsequently, the results of VA_S_ and VA_C_ were corrected for the calculated real test distance:

$corrected {VA}_{S/C} [logMAR]=log (\frac{{(10}^{VA \left[ logMAR \right]}) * 5 m}{real viewing distance \left[ m \right]})$.

Correction of distance variability for VA data was conducted using IGOR (IGOR Pro; WaveMetrics, Portland, OR, USA) to correct for the actual viewing distance for the visual acuity values collected.

**Sample size calculation:**

The data presented were obtained during the baseline measurements of a longitudinal study examining the effects of a multimodal vs. unimodal exercise intervention on visual function, cognitive performance as well as brain connectivity in glaucoma and healthy elderly participants. Using G*Power (3), a sample size calculation was conducted for a repeated measures analysis of variance (RM-ANOVA) with two groups (glaucoma patients, healthy elderly) and the effect of different exercise paradigms. Based on related studies of gait velocity in glaucoma (4,5), a total sample size of 26, 13 in each group, was required to obtain a medium effect size (f = 0.25) at a significance level of 0.05 and a power of 0.95.

**Results**

***a) Impact of TM-walking speed on viewing distance.***

i) BCVA

For an initial assessment of the relevance of the distance changes induced by different TM-walking speeds, we compared the S_0_ condition to S_self_ and S_3.5_ (supplementary Figure 1A and supplementary Table 2). For both groups (no main effect of GROUP, p = 0.152), the viewing distance was significantly greater during S_self_ (p < 0.001) and S_3.5_ (p < 0.001) compared to the S_0_ condition by 15.6 ± 1.6 cm and 14.1 ± 1.7 cm, respectively. A slightly larger distance was evident in the first repetition than in the second for S_self_ (p < 0.001) and S_3.5_ (p = 0.016) by 3.83 ± 0.89 cm and 2.59 ± 1.03 cm. Taken together, the observed effects of different TM-walking speeds on viewing distance were small compared to the actual viewing distance, i.e., < 5% (16 cm/500 cm * 100%). Still, residual potentially confounding effects on the accuracy of the BCVA values were taken into account in the analysis. As the systematic effects of different TM-walking speeds on viewing distance were determined with the Vivior^®^ sensor, they were used to correct the BCVA values for both VA_s_ and VA_c_ during TM-walking, by re-calculating the logMAR values for the actual viewing distance of each individual.

ii) VF

At first, we assessed the differences between VF-MD during on/off distance controls operated by Ocusweep^®^ (6) and demonstrated significantly better VF values for the latter condition (p = 0.003). Importantly, the size of this effect was small (0.23 ± 0.08 dB) and the relative intra-session reliability of both measurements was high (ICC = 0.97), which underlines the reliability of the Ocusweep^®^ without distance monitoring (Supplementary Figure 1B i, ii).

***(b) Correlation of structural and functional indices****.* For further exploration, we tested the dependence of the functional measures, i.e., VA, CS, and VF of S_0_ condition, with structural measures provided by the OCT, i.e., pRNFL and mGCLIPL (average across both eyes). For this analysis, the VF-MD of the binocular measurement with Ocusweep^®^ with activated distance-control was utilized. Correlations were significant for VF-MD vs pRNFL thickness (r = -0.362, p = 0.024), mGCLIPL volume (r = -0.429, p = 0.006) and vs CS (r = -0.381, p = 0.024, see supplementary figure 2 and Supplementary Table 3).

To check whether worse OCT or VF-MD measures were associated with poorer visual performance during TM-walking, their correlation with visual function loss [VAS, VAC, CS, MD] was determined, but no significant associations were found (Supplementary Table 3 and figure 2). This might be due to the small range of vision loss in general (see Supplementary Figure 2), due to the early-stage nature of the majority of glaucoma patients.

Supplementary Table 1. Descriptive data and group differences

|  | Group | | | |  |
| --- | --- | --- | --- | --- | --- |
|  | **Healthy controls (n = 30)** | | **Glaucoma (n = 18)** | | **p-value** |
| Sex^+^ | m | 13 | m | 9 | 0.768 |
|  | f | 17 | f | 9 |  |
|  | **mean ± SEM [median \| range]** | | **mean ± SEM [median \| range]** | |  |
| Age | 71 ± 0.93 | | 71 ± 1.26 | | 0.819 |
| BCVA | -0.1 ± 0.02 [-0.1 \| 0.4] | | -0.06 ± 0.03 [-0.05 \| 0.5] | | 0.110 |
| pRNFL_R | 91 ± 2.24 | | 77 ± 3.16^#^ | | 0.002 |
| pRNFL_L | 89 ± 1.97 | | 76 ± 3.62 | | 0.002 |
| pRNFL_RL | 90.25 ± 2.06 | | 75.97 ± 2.83^#^ | | < 0.001 |
| mGCL_R | 1.03 ± 0.02 | | 0.91 ± 0.02^#^ | | < 0.001 |
| mGCL_L | 1.02 ± 0.02 | | 0.87 ± 0.03 | | < 0.001 |
| mIPL_R | 0.86 ± 0.01 | | 0.78 ± 0.01^#^ | | < 0.001 |
| mIPL_L | 0.85 ± 0.01 | | 0.77 ± 0.02 | | 0.001 |
| mGCLIPL_RL | 1.88 ± 0.03 | | 1.65 ± 0.03^#^ | | < 0.001 |
| MD_R | [0.59 \| 5.09] | | [-0.95 \| 25.97] | | 0.036 |
| MD_L | [0.15 \| 5.92] | | [-1.04 \| 22.00] | | 0.047 |
| MD binocular | [-0.7 \| 3.3] | | [0.4 \| 9.6] | | 0.009 |
| ^+^ Chi-square test  ^#^ OCT measurement of one right eye was not possible, n = 17.  For values without normal distribution, Mann-Whitney-U test was used for analysis.  SEM = standard error of the mean, BCVA = best corrected visual acuity [logMAR], pRNFL_R/L= peripapillary retinal nerve fiber layer (pRNFL) thickness [µm], pRNFL = averaged peripapillary retinal nerve fiber layer thickness [µm], mGCL_R/L = volume of macular ganglion cell layer [mm^3^], mIPL_R/L = volume of macular inner plexiform layer [mm^3^], GCLIPL = sum of ganglion cell layer (mGCL) and inner plexiform layer (mIPL) thicknesses [mm^3^] (the GCIPL is a robust measure to reflect activities in both GCL and IPL layers and it is adopted in the literature as a surrogate measure of ganglion cell damage in glaucoma), MD_R/L = mean deviation [dB], measured with Humphrey Field Analyzer (negative MD indicates VF loss), MD_binocular = mean deviation [dB], measured binocular with Ocusweep^®^ (positive MD indicates VF loss) and activated distance control | | | | | |

Supplementary Table 2. Results of ANOVA and post-hoc analyses for distance variability

|  | RM-ANOVA | | | | Post-hoc | | |
| --- | --- | --- | --- | --- | --- | --- | --- |
|  | **Factors** | **F** | **p** | **ⴄ^2^** | **t-tests** | **Mean diff.** | **p** |
| *Viewing distance*  *[mm]* | Group | F(1,46) = 2.1 | 0.152 | 0.04 | - | - | - |
|  | Speed | F(1.3,61.4) = 73.7 | **< 0.001** | 0.62 | S_0_ vs S_Self_  S_0_ vs S_3.5_  S_Self_ vs S_3.5_ | -155.9  -141.2  14.7 | **< 0.001**  **< 0.001**  0.176 |
|  | Repetition | F(1,46) = 9.4 | **0.004** | 0.17 | - | - | - |
| *Visual field-MD static [dB]* | Group | F(1,46) = 10.0 | **0.003** | 0.18 | - | - | - |
|  | Distance control | F(1,46) = 9.5 | **0.003** | 0.17 | With vs without | 0.0^#^ | **0.006^#^** |
| Significant results are highlighted in bold.  ^#^ Wilcoxon test, with difference of medians  Mean diff. = mean difference | | | | | | | |

Supplementary Table 3. Correlations of visual performance and visual loss with OCT-values and visual field.

|  | pRNFL [µm] | | mGCLIPL [mm^3^] | | MD [dB] | | |
| --- | --- | --- | --- | --- | --- | --- | --- |
|  | Correlation coefficient r | p-value | Correlation coefficient r | p-value | Correlation coefficient r^#^ | p-value^#^ | |
| Visual performance |  |  |  |  |  |  |  |
| VA_S_ [logMAR] | -0.049 | 0.741 | -0.217 | 0.144 | 0.247 | 0.090 |  |
| VA_C_ [logMAR] | -0.120 | 0.423 | -0.237 | 0.109 | 0.227 | 0.121 |  |
| CS [logCS] | 0.056 | 0.98 | 0.254 | 0.23 | -0.381 | **0.024** |  |
| MD [dB] | -0.362 | **0.024** | -0.429 | **0.006** | - | - |  |
| Visual loss |  |  |  |  |  |  |  |
| VA_S_ loss [logMAR] | 0.02 | 0.896 | -0.022 | 0.884 | 0.115 | 0.437 | |
| VA_C_ loss [logMAR] | -0.120 | 0.422 | 0.092 | 0.540 | 0.072 | 0.628 | |
| CS loss [logCS] | -0.038 | 0.800 | -0.069 | 0.644 | 0.069 | 0.640 | |
| MD loss [dB] | 0.011 | 0.941 | -0.190 | 0.207 | 0.255 | 0.084 | |
| ^#^ The analysis was performed with Spearman correlation due to non-normal distribution.  Significant correlations are highlighted in bold.  For abbreviations see supplementary Table 1. | | | | | | | |

|  |
| --- |
|  |
| 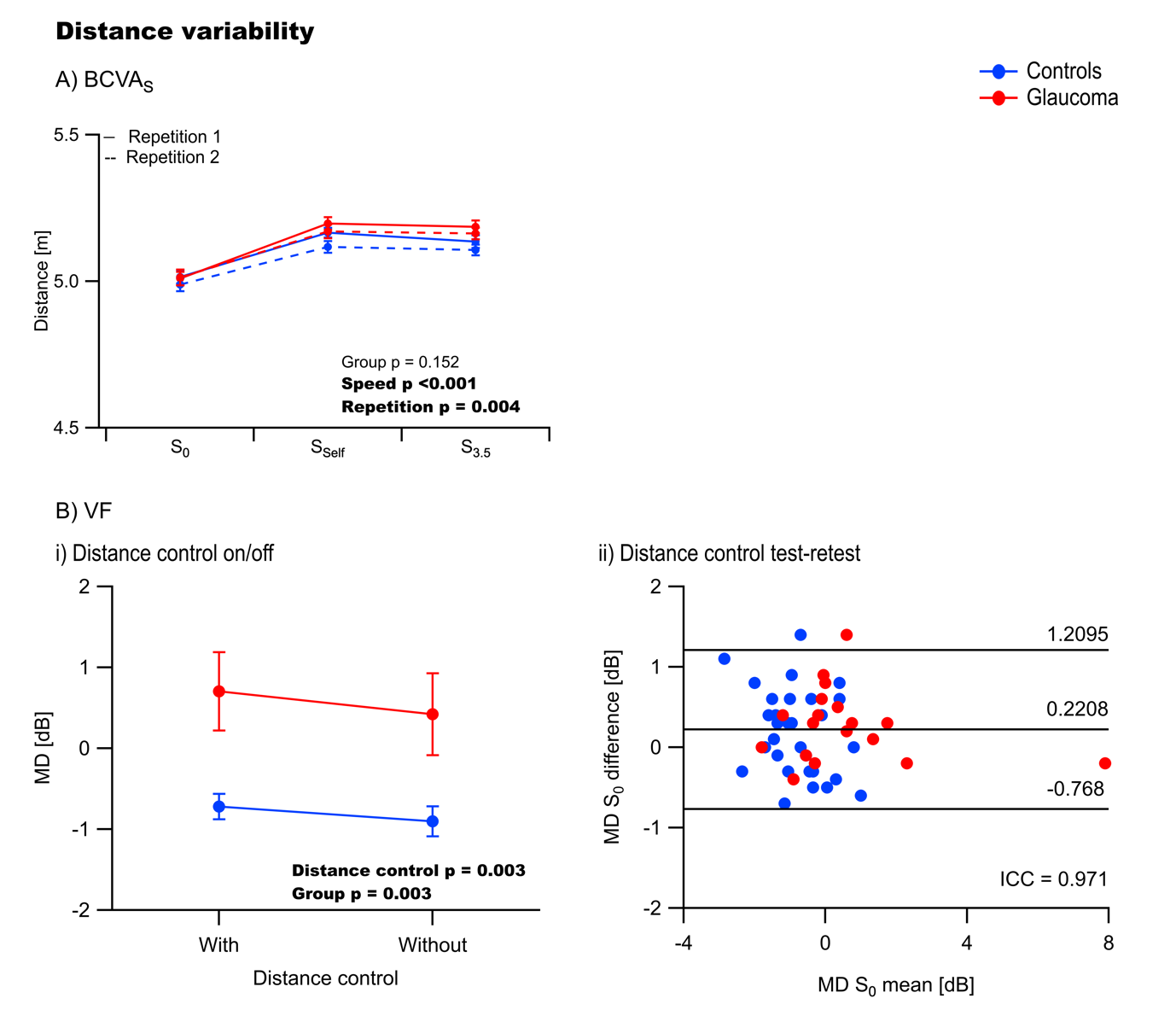 |
| Supplementary Figure 1. Impact of different TM-walking speeds on viewing distance  (A) BCVA. (i) Effect of TM-walking speeds on viewing distance; viewing distances were increased during TM-walking compared to standing and for the 1st compared to 2nd repetition.  (B) Visual field (VF). (i) Effect of distance control; VF was better without distance control in the static measurements. (ii) Bland-Altman diagram of test-retest repeatability for static VF measurements; VF with and without distance control were similar (high intraclass correlation coefficient). BCVA = best corrected visual acuity, ICC = intraclass coefficient. |


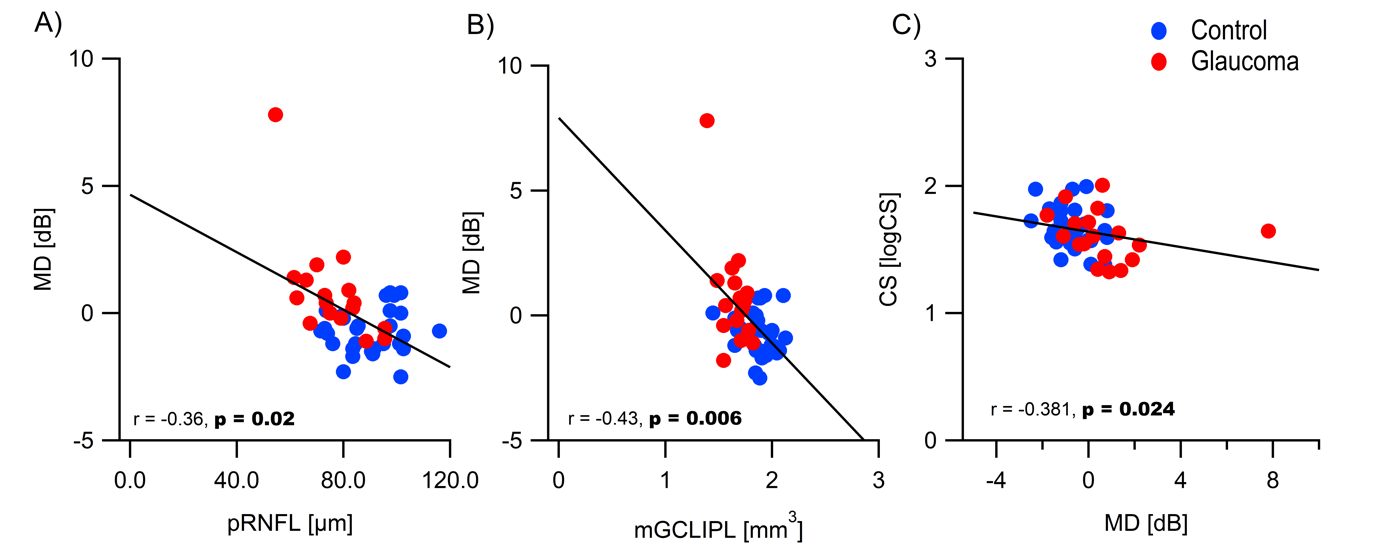


Supplementary Figure 2. Functional and structural correlates.

(A) Correlation of visual field (VF) and peripapillary retinal nerve fiber layer (pRNFL): higher values of mean deviation correlate with lower values of peripapillary retinal nerve fiber layer thickness.

(B) Correlation of VF and macular ganglion cell inner plexiform layer (mGCLIPL): higher values of mean deviation correlate with lower values of the volume of macular ganglion cell and inner plexiform layer.

(C) Correlation of CS and MD: higher values of CS correlate with lower values of MD.

For details see text, for abbreviations see supplementary Table 1.

Literature Cited

1. Vivior AG, editor. Die Innovation: Objektive Messdaten für personalisierte Lösungen 2020.

2. Vivior AG. VIVIOR - Wie Sie digitalen Sehstress vermeiden [cited 2023 Apr 6]. Available from: URL: <https://vivior.com/>.

3. Faul F, Erdfelder E, Lang A-G, Buchner A. G*Power 3: a flexible statistical power analysis program for the social, behavioral, and biomedical sciences. Behavior research methods. 2007;39(2):175-191.

4. Gomes HdA, Moreira BdS, Sampaio RF, et al. Gait parameters, functional mobility and

fall risk in individuals with early to moderate primary open angle glaucoma: a cross-sectional study. Brazilian journal of physical therapy. 2018;22(5):376-382.

5. Lee H-S, Lee K-J, Kim J-L, Leem H-S, Shin H-J, Kwon HG. Gait characteristics during crossing over obstacle in patients with glaucoma using insole foot pressure. Medicine. 2021;100(32):e26938.

6. Ocuspecto Oy, editor. Ocusweep® User Guide: Including service and maintenance instructions 2021.
